# Innovative strategies for post-stroke depression: integrating traditional Chinese medicine with neurobiological insights, including the gut-brain axis

**DOI:** 10.3389/fphar.2025.1539357

**Published:** 2025-06-03

**Authors:** Lin Zhu, Ruina Han, Linxia He, Bingfa Pan, Weijie Zhong, Yi Li, Xinru Liu

**Affiliations:** ^1^ Institute of Translational Medicine, Shanghai University, Shanghai, China; ^2^ Department of Neurosurgery, Shanghai Ninth People’s Hospital, Shanghai Jiao Tong University School of Medicine, Shanghai, China; ^3^ Shuguang Hospital, Shanghai University of Traditional Chinese Medicine, Shanghai, China

**Keywords:** post-stroke depression (PSD), traditional Chinese medicine (TCM), gut-brain axis, neuroinflammation, neurotransmitter

## Abstract

Post-stroke depression (PSD) is a debilitating condition affecting more than one-third of stroke survivors, leading to significant impairments in mood, cognitive function, and overall quality of life. While conventional treatments like selective serotonin reuptake inhibitors (SSRIs) are commonly used, their efficacy is often limited, and they are associated with adverse side effects. Emerging research underscores the critical roles of neuroinflammation, neurotransmitter imbalances, and disruptions in the gut-brain axis in the development and progression of PSD, suggesting that targeting these pathways could lead to more effective therapeutic outcomes. Traditional Chinese Medicine (TCM) presents a promising multi-faceted approach, addressing these complex biological mechanisms by regulating neurotransmitter systems, modulating immune responses, and restoring gut microbiota balance. Key herbs such as *Salvia miltiorrhiza* Bunge (Lamiaceae; Dan Shen) and *Bupleurum chinense* DC. (Apiaceae; Chai Hu) have shown significant potential in modulating neurotransmitter levels, reducing neuroinflammation, and providing neuroprotection. Additionally, TCM formulations like Chaihu Shugan Powder (CSP) and Shugan Jieyu Capsules (SG) further enhance these effects by promoting gut microbiota homeostasis and restoring metabolic balance. This review delves into the biological mechanisms underlying PSD, with a particular focus on neuroinflammation, neurotransmitter dysregulation, and gut-brain axis dysfunction. It also explores the potential of integrating TCM with advanced multi-omics technologies—such as metabolomics, metagenomics, and transcriptomics—to develop personalized treatment strategies for PSD. By combining the holistic principles of TCM with modern Western medicine and cutting-edge omics technologies, this integrative approach offers a comprehensive framework for managing PSD, with the potential to significantly improve recovery outcomes and enhance the quality of life for stroke survivors.

## 1 Introduction

Stroke, a major cause of disability and mortality worldwide, occurs when cerebral blood flow is interrupted, leading to neuronal damage and functional impairment (Hankey, 2014; Campbell et al., 2019). Among survivors, over one-third develop post-stroke depression (PSD), characterized by persistent low mood, reduced interest in activities, and cognitive decline (Villa et al., 2018; Cai et al., 2019; Medeiros et al., 2020; Guo et al., 2022). PSD prevalence can reach 31% within 5 years post-stroke, significantly hindering recovery and posing a public health burden (Carnes-Vendrell et al., 2019; Lanctot et al., 2020; Frank et al., 2022)

PSD is frequently associated with gastrointestinal dysfunction, reflecting the intricate relationship between the nervous and gastrointestinal systems (Frank et al., 2022). The gut microbiota, a key regulator of immune function, metabolism, and brain activity, plays a crucial role in stroke recovery. Stroke-induced dysbiosis not only alters the production of metabolites like Trimethylamine-N-oxide (TMAO) and Short-chain fatty acids (SCFAs) but also triggers chronic inflammation and neurotransmitter imbalances (e.g., serotonin and dopamine), exacerbating depressive symptoms. Additionally, inflammatory pathways activated by dysbiosis impair neuroprotection and brain recovery, establishing a key link in the development of PSD (Zhu et al., 2016; Lee et al., 2020). Dysregulated microbiota further influences mood by modulating neurotransmitter pathways (Liang S. et al., 2018; Waclawikova and El Aidy, 2018; Ge et al., 2021; Bai et al., 2022). These findings highlight the gut-brain axis as a promising therapeutic target in PSD (Jiang et al., 2021; Zhong et al., 2022).

Despite the widespread use of selective serotonin reuptake inhibitors (SSRIs) and serotonin-norepinephrine reuptake inhibitors (SNRIs) for PSD treatment, these drugs are often limited by side effects, such as insomnia and gastrointestinal disturbances, which complicate recovery (Mikami et al., 2011; Mortensen et al., 2013). Furthermore, many patients exhibit resistance to these therapies, with trials showing no significant differences between antidepressants and placebos in symptom relief (Robinson et al., 2000; Savadi Oskouie et al., 2017).These challenges highlight the need for alternative, multi-targeted approaches to PSD management.

Traditional Chinese Medicine (TCM) has demonstrated therapeutic efficacy in other neuropsychiatric conditions, including anxiety, depression, and cognitive impairment, by modulating neurotransmitter systems and immune responses (Guo et al., 2021). These findings suggest that TCM may offer unique advantages in managing PSD through its multi-targeted approach. By integrating multiple herbal components tailored to individual symptoms, TCM formulations modulate neurotransmitter levels, immune responses, and gut microbiota composition, addressing the diverse pathways involved in PSD pathogenesis (Li et al., 2020; Li et al., 2022). Compared to single-target pharmacotherapies, TCM’s holistic approach offers a broader framework for managing PSD, targeting both emotional disturbances and physical dysfunctions.

**FIGURE 1:**
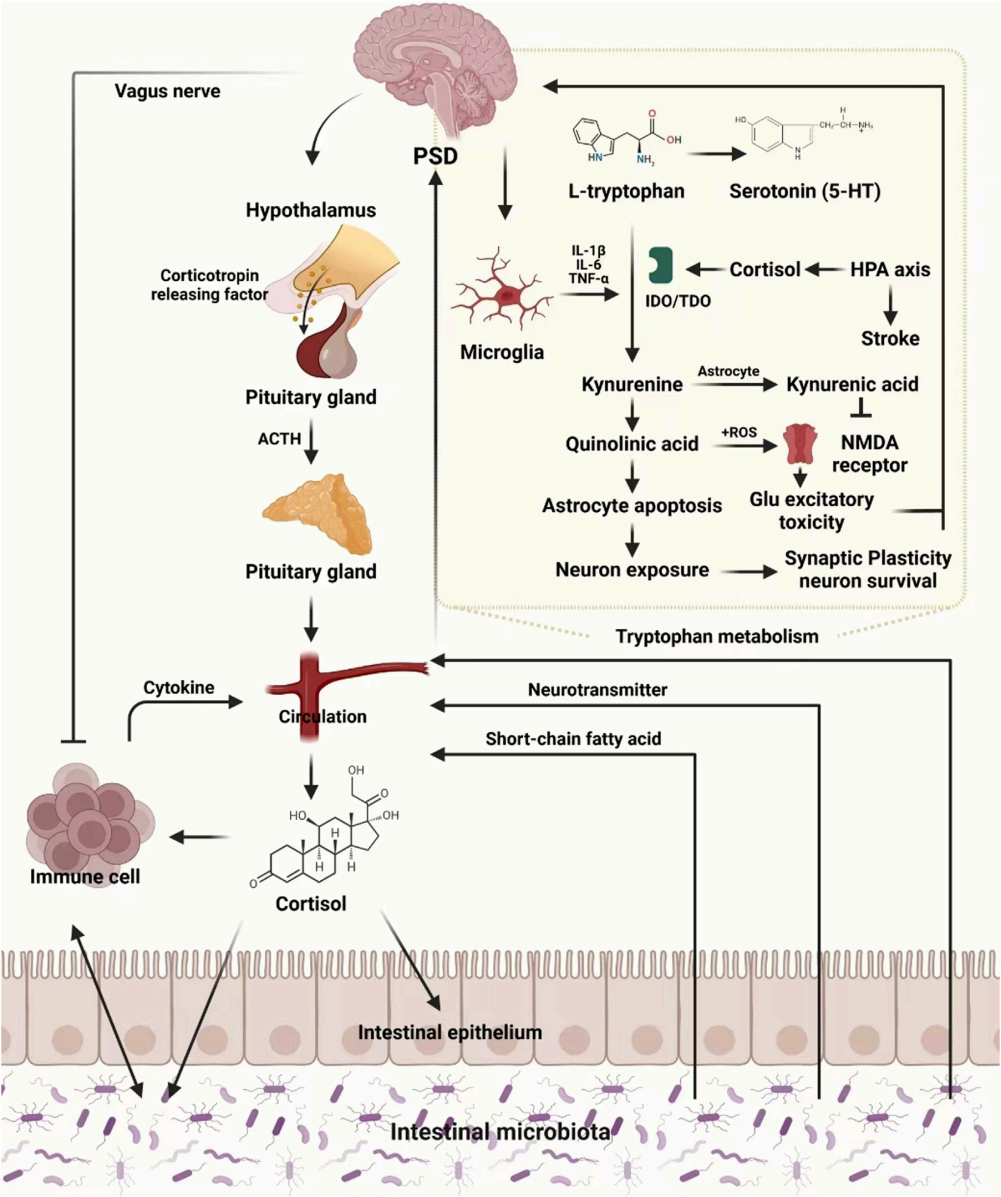
Molecular mechanisms underlying PSD.

However, the use of TCM in PSD management remain under explored. Integrating TCM with Western medicine could bridge the gaps in existing treatments, offering a complementary strategy to address the complex pathophysiology of PSD. This review aims to analyze the biological mechanisms underlying PSD and explore the therapeutic potential of TCM interventions targeting the gut-brain axis and neuroinflammation. By integrating insights from both Western medicine and TCM, this work aims to inform the development of innovative therapeutic strategies for improving the quality of life in PSD patients (Figure 1).

## 2 TCM symptoms related to PSD

In TCM, PSD is conceptualized as a dual phenomenon encompassing both “Stroke” and “Depression.” Stroke leads to qi and blood stagnation, blocking cerebral circulation and causing symptoms like paralysis, speech impairment, and numbness. Depression arises from qi stagnation, heart and spleen deficiencies, and phlegm-blood accumulation. Emotional disturbances block the flow of qi, leading to liver qi stagnation, which manifests as low mood, chest tightness, and pain. Prolonged stagnation can cause phlegm obstruction, resulting in palpitations, excessive phlegm, and chest constriction. Unresolved emotional stress worsens heart and spleen deficiencies, causing fatigue, appetite loss, and insomnia.

The core pathological mechanism of PSD involves blocked qi flow and impaired circulation (Huang et al., 2018). Stroke-induced stagnation disrupts these pathways, and depression intensifies the imbalance, creating a vicious cycle that worsens physical and emotional symptoms. Key organs include the liver, kidney, heart, and spleen. The liver, crucial for regulating blood, qi, and emotions, plays a central role, as impaired liver function can aggravate depressive symptoms. Liver qi stagnation is especially critical, forming a cycle where emotional distress worsens stagnation, which deepens depression.

Understanding Qi deficiency and Yin-Yang (nourishing-activating) imbalances in PSD is crucial for effective treatment. Qi deficiency, linked to decreased energy metabolism, leads to neuroinflammation and immune dysfunction, exacerbating depressive symptoms. In PSD, this results in the increased release of pro-inflammatory cytokines (e.g., IL-6, TNF-α), damaging neurons and impairing synaptic function (Yan, 2018; Fu et al., 2021; Feng et al., 2022).Yin-Yang imbalances further disrupt neurotransmitter regulation and the HPA axis. Yin and Yang play opposite roles in regulating the body. Excessive Yang can overactivate the HPA axis, while Yin deficiency impairs neurotransmitter production and mood regulation, worsening depressive symptoms in PSD patients (Ding et al., 2024).

The interaction between Qi deficiency and Yin-Yang imbalances may also affect the gut-brain axis. Qi deficiency is associated with gut dysbiosis, where pathogenic bacteria increase and beneficial bacteria decrease, leading to systemic inflammation and neuroinflammation. Yin deficiency may exacerbate these issues, worsening neurotransmitter imbalances and inflammation (Jiang et al., 2023). Herbal formulas (e.g., Chaihu Shugan Powder (CSP), *Salvia miltiorrhiza* Bunge (Lamiaceae; Dan Shen), *Astragalus membranaceus* Fisch. ex Bunge (Fabaceae; Huang Qi) targeting these TCM syndromes can regulate these molecular pathways and improve clinical outcomes for PSD patients (Kwon et al., 2019).

## 3 The underlying molecular mechanism in PSD

**FIGURE 2:**
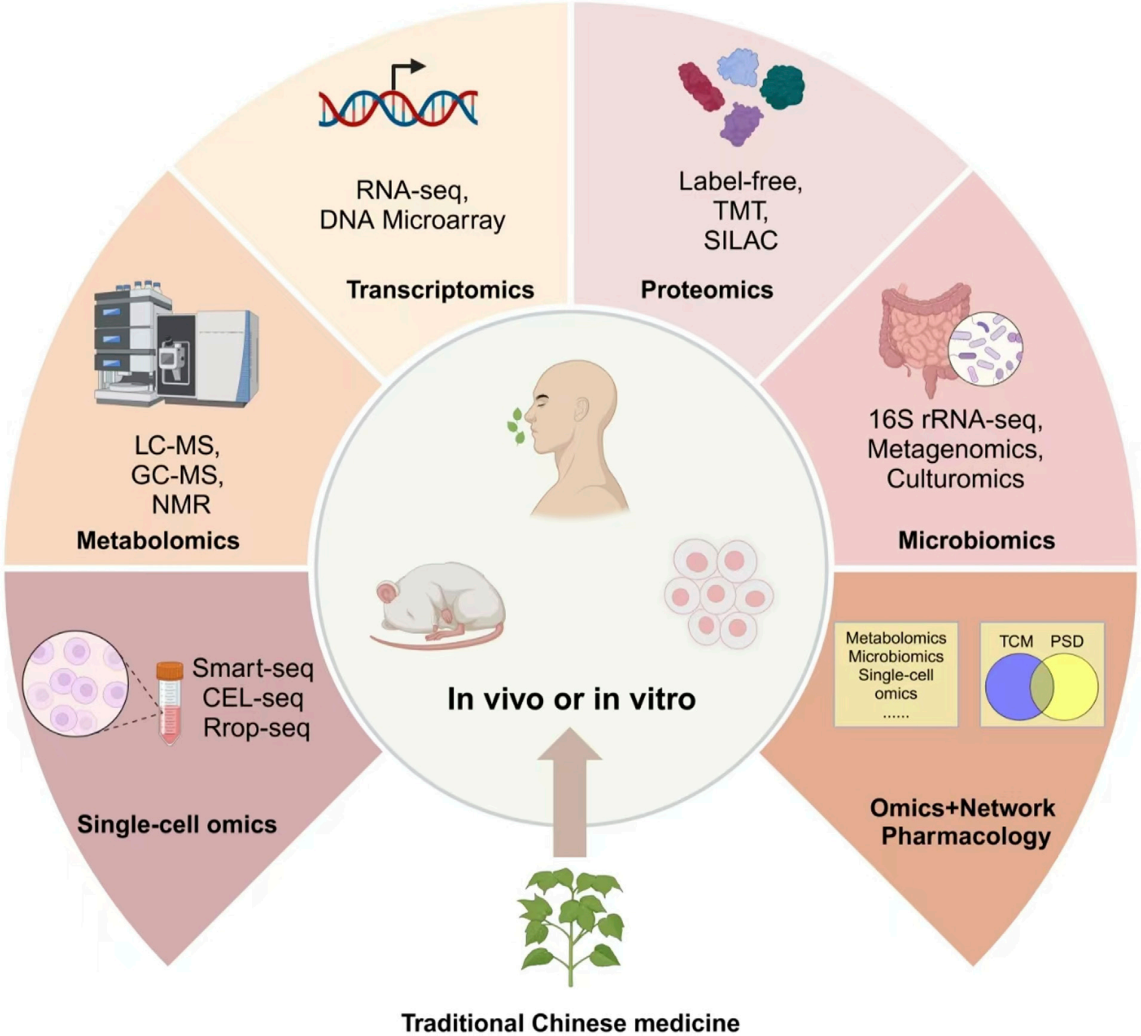
Multi-omics approaches to optimize TCM interventions.

The pathogenesis of PSD remains complex and not fully understood, with research indicating contributions from stroke lesion locations, genetic predispositions, neurotransmitter imbalances, neuroendocrine alterations, neurotrophic factors, and neuroinflammatory processes (Figure 2). This section focuses on the molecular pathways most relevant to TCM interventions, including BDNF regulation, HPA axis modulation, neurotransmitter balance, and neuroinflammatory suppression. In addition, for the sake of completeness, all other relevant molecular mechanism pathway diagrams are presented in Table 1.

**TABLE 1:** The underlying molecular mechanism in PSD.

Mechanism type	Molecular pharmacological mechanism	Research object	Stroke Type/Mode	Depression assessment	Relevant result	References
Stroke lesion site	Frontal Lobe	PSD patient	Ischemic stroke	DSM-IV and HAMD	PSD affects mood through the brain network of the prefrontal-limbic circuit.	Shi et al. (2017)
	Amygdala-cortical FC	PSD patient	Ischemic stroke	DSM-IV and HAMD	Hyperconnectivity between the amygdala, default mode network, and salience network might be related to depressive symptoms.	Fan et al. (2023b)
	DLPFC	PSD patient	Ischemic stroke	SAS, HAMD and CES-D	Lesion locations of PSD mapped to the depression circuit centered by left DLPFC.	Zhang et al. (2019)
	Left middle frontal gyrus	PSD patient	Ischemic stroke	DSM-IV and HAMD	The hypoactivity in the left IFG and DLPFC as well as the reduced prefrontal inhibition to the limbic system in PSD patients.	Hong et al. (2020)
Genetic	5-HTTLPR and STin2 VNTR	PSD patient	Stroke	GDS and DSM -IV	Individuals with the 5-HTTLPR s/s genotype had 3-fold higher odds of PSD compared with l/l or l/xl genotype carriers. Participants with the STin2 9/12 or 12/12 genotype had 4-fold higher odds of PSD compared with STin2 10/10 genotype carriers.	Kohen et al. (2008)
	Apo E	PSD patient	Stroke	HAMD	Delayed P300, elevated serum ApoE and decreased monocyte ApoE expression are associated with PSD.	Zhang et al. (2013)
	p11/tPA/BDNF pathway	PSD patient	Acute ischemic stroke	DSM -IV and HAMD	TrkB gene, BDNF and TrkB haplotypes, and gene-gene interactions between p11, tPA and BDNF are all associated with PSD.	Liang et al. (2018a)
	HTR3D and NEUROG3	PSD patient	Stroke	DSM -IV and HAMD	HTR3D and NEUROG3 were linked with the susceptibility of PSD and PIK3C2B with stroke in the Chinese Han population.	Fuying et al. (2019)
Neurotransmitter	Monoamine neurotransmitter	Rats	mPFC and microinjection of ET-1	EPM	Abnormal expression of serotonin in mPFC, nucleus accumbens, septum, hippocampus, BLA, and dorsal raphe.	Zahrai et al. (2020)
		PSD patient	Ischemic stroke	HAMD-17	PSD is positively correlated with the SP value but negatively correlated with the CCK-8 and 5-HT.	Zhang et al. (2023a)
	Glutamate-Mediated Excitotoxicity	PSD patient	Acute ischemic stroke	HAMD-17	Plasma glutamate and glutamate oxaloacetate transaminase levels were strongly associated with the development of PSD within 3 months of admission.	Cheng et al. (2014)
		Rats	MCAO	SPT	Elevated glutamate levels in the central nervous system of rats.	Frank et al. (2019)
		PSD patient	Acute ischemic stroke	BDI and HAMD-17	An association between the early-onset PSD and a low plasma glutamate level following acute ischemic stroke	Geng et al. (2017)
Neuroendocrine	HPA	PSD patient	Acute ischemic stroke	DSM-IV and HAMD-17	The levels of both IL-6 and cortisol were increased in the sera of PSD patients.	Zhang et al. (2016)
	CB1 and CB2	Rats	MCAO+CUMS	SPT and OFT	CB1 or CB2 receptor stimulation prevents post-stroke depression.	Wang et al. (2016)
Neurotrophic factors	BDNF	Rats	MCAO+CUMS	OFT, FST and SPT	The unbalance between BDNF and proBDNF in the ischemic hippocampus played an important role in the pathogenesis of PSD.	Luo et al. (2019a)
	GDNF	PSD patient	Ischemic stroke	DSM-IV, HAMD and MMSE	GDNF may serve as a potential biomarker for differential diagnosis of PSD patients.	Zhang et al. (2017)
	IGF-1	PSD patient	Acute ischemic stroke	DSM-III-R and HAMD	Low serum IGF-1 levels at admission are associated with a high risk of developing PSD	Zhang et al. (2018)
	IGF-1	PSD patient	Ischemic stroke	HAMD and MMSE	Patients with rs9282715 T allele of IGF-1R may have PSD susceptibility	Yue et al. (2023)
Neuroinflammation	TNF-α and IL-1β	PSD patient	Acute ischemic stroke	DSM-IV	TNF-α and IL-1β serum levels play regarding the risk of PSD.	Kim et al. (2017)
	IL-10	PSD patient	Acute ischemic stroke	DSM-V and HAMD	Lower IL-10 levels may be used to predict PSD.	Chi et al. (2021)
	IL-1β	PSD patient	Acute ischemic stroke	DSM-V and HAMD	IL-1β is strongly associated with PSD at 6 months after stroke.	Yi et al. (2021a)
	IL-6 and IL-18	PSD patient	Stroke	DSM-IV	Higher levels of IL-6 and IL-18 are related to PSD at 2 weeks and 1 year after stroke.	Kang et al. (2016)
	IL-18	Rats	MCAO+Stress	TST and FST	Increased IL-18 level in the amygdala mediated depression-like behaviors in a mouse PSD model.	Wu et al. (2020)
	GDF-15	PSD patient	Acute ischemic stroke	Beck Depression Inventory Fast Screen BDI-FS)	GDF-15 serum levels at admission are associated with depression later developed in patients with ischemic stroke.	Lu et al. (2020b)
	GDF-15	PSD patient	Acute ischemic stroke	HRSD-24	GDF‐15 can be a valuable prognostic biomarker for PSD.	Zang et al. (2022)
	GDF-15, aCL, aPS and MMP-9	PSD patient	Acute ischemic stroke	HRSD-24	Combination of GDF-15, aCL, aPS and MMP-9 substantially improved the risk prediction of depression at 3 months after ischemic stroke.	Che et al. (2021)
	Hs-CRP and HCY	PSD patient	Acute ischemic stroke	DSM-IV and HAMD-17	elevated serum levels of Hs-CRP and HCY were associated with the risk of developing PSD 1 year after the stroke onset.	Cheng et al. (2018)

### 3.1 Overview of brain lesions, genetic factors, and glutamate excitotoxicity

Stroke-related lesions in areas such as the thalamus, basal ganglia, and prefrontal cortex impair neurotransmitter systems, contributing to depressive symptoms (Shi et al., 2017). For instance, left frontal lobe lesions correlate with serotonin and norepinephrine depletion, exacerbating emotional dysregulation (Klingbeil et al., 2021). Functional imaging studies reveal disrupted amygdala-prefrontal cortex connectivity, further linking brain damage to depressive symptoms (Zhang et al., 2019; Fan Y. et al., 2023).

Genetic predispositions also influence PSD vulnerability. Polymorphisms in 5-HTTLPR, MTHFR, and ApoE have been associated with a higher risk of PSD (Kohen et al., 2008; Zhang et al., 2013). Additionally, variations in the p11/tPA/BDNF pathway affect depressive outcomes following stroke (Liang J. et al., 2018).

Glutamate excitotoxicity triggered by ischemia and hypoxia can cause neuronal damage and synaptic dysfunction. Elevated glutamate levels in cerebrospinal fluid have been associated with PSD symptoms, though plasma concentrations may vary (Cheng et al., 2014; Geng et al., 2017). TCM therapies, such as *S. miltiorrhiza* Bunge (Lamiaceae; Dan Shen), indirectly mitigate excitotoxicity by promoting synaptic plasticity and neurotransmitter balance.

### 3.2 Brain-derived neurotrophic factor (BDNF) regulation

BDNF plays a crucial role in synaptic plasticity, neuronal survival, and emotional regulation. Stroke impairs BDNF signaling, disrupting neurogenesis and axonal regeneration, which increases the risk of developing PSD. BDNF exerts its effects through p75 neurotrophin receptor (p75NTR) and tropomyosin receptor kinase B (TrkB). However, the imbalance between BDNF and proBDNF promotes neuronal apoptosis, as proBDNF activates the RhoA-JNK signaling pathway, inhibiting synaptic recovery. TCM interventions, such as *Bupleurum chinense* DC. (Apiaceae; Chai Hu) and *S. miltiorrhiza* Bunge (Lamiaceae; Dan Shen), enhance BDNF levels through the ERK-CREB-BDNF pathway, promoting emotional recovery (Yang et al., 2021). Maintaining the BDNF/proBDNF balance is essential for neuroprotection and functional recovery. Both aerobic exercise and TCM therapies have been found to enhance this balance, promoting axonal regeneration and improving mood regulation in PSD patients (Luo L. et al., 2019).

Additional neurotrophic factors, such as insulin-like growth factor-1 (IGF-1) and glial cell line-derived neurotrophic factor (GDNF), also support neuronal recovery. GDNF promotes axon regeneration and enhances brain tissue plasticity (Beker et al., 2020; Zhang et al., 2018). Clinical studies have further shown that GDNF levels are negatively correlated with Hamilton Depression Rating Scale (HAMD) scores, suggesting that GDNF may serve as a diagnostic marker for PSD (Zhang et al., 2017). Variants in the IGF-1R gene, particularly the T allele at the s9282715 locus, have also been linked to an increased risk of PSD (Yue et al., 2023).

### 3.3 HPA axis modulation

The HPA axis plays a key role in regulating the stress response, emotional stability, and immune function. Stroke acts as both a direct and indirect stressor, disrupting the HPA axis and leading to excessive glucocorticoid (GC) production, primarily cortisol. Elevated cortisol levels have been strongly linked to depressive symptoms in PSD patients (Zhanina et al., 2022; Zhang et al., 2016). Dysregulation of the HPA axis contributes to persistent stress responses, immune dysfunction, and inflammation, further exacerbating depressive behavior (Wang et al., 2016).

Following a stroke, the hippocampus and adjacent brain regions send signals to the hypothalamus, stimulating the release of corticotropin-releasing hormone (CRH). This triggers the pituitary gland to release adrenocorticotropic hormone (ACTH), which, in turn, stimulates the adrenal cortex to produce glucocorticoids. While glucocorticoids regulate metabolism and immune response, chronic overproduction disrupts emotional regulation and impairs neuronal function by affecting neurogenesis and neurotransmitter levels (Zhou et al., 2022).

TCM interventions have shown potential in modulating the HPA axis. Shugan Jieyu Capsule (SG) and *Glycyrrhiza uralensis* Fisch. ex DC. (Fabaceae; Gan Cao) help restore cortisol homeostasis by suppressing excessive GC production and reducing neuroinflammation. This modulation of the HPA axis has been associated with improved emotional regulation and mood stability in PSD patients. Activation of CB1 and CB2 receptors has also been shown to mitigate depressive-like behavior by regulating HPA axis activity in rodent models (Wang et al., 2016; Zhang S. et al., 2023).

### 3.4 Neurotransmitter imbalances

Neurotransmitter imbalances, particularly in 5-HT, DA, and NE, play a critical role in the development of PSD. Stroke lesions in regions such as the basal ganglia, prefrontal cortex, and thalamus impair neurotransmitter synthesis, release, and reuptake, disrupting emotional regulation and cognition. Left frontal lobe damage is especially associated with significant 5-HT and NE reduction, increasing depression risk.

The monoaminergic system is vital for regulating mood, sleep, and cognition. Stroke disrupts this system, limiting neurotransmitter release and axonal regeneration. For example, reduced 5-HT levels in the frontal lobe and hippocampus correlate with depressive behaviors (Zahrai et al., 2020), while disruptions in the GR/ERβ/TPH2 pathway impact serotonin synthesis and depressive symptoms (Zhang X. et al., 2023).

TCM interventions restore neurotransmitter balance. *Salvia miltiorrhiza* Bunge (Lamiaceae; Dan Shen) boosts serotonin and dopamine levels, enhancing mood and cognition, while *Bupleurum chinense* DC. (Apiaceae; Chai Hu) modulates neurotransmitter activity through the ERK-CREB-BDNF pathway, promoting emotional stability (Yang et al., 2021; Zhang S. et al., 2023). Though glutamate excitotoxicity contributes to stroke-related neuronal damage, TCM focuses on monoamine regulation to improve synaptic plasticity. Early interventions targeting neurotransmitter imbalances, such as restoring 5-HT levels, show promise for improving PSD outcomes (Cheng et al., 2014; Geng et al., 2017).

### 3.5 Neuroinflammatory processes

Neuroinflammation plays a crucial role in the pathogenesis of PSD, contributing to neuronal damage, synaptic dysfunction, and emotional disturbances. Stroke induces the release of pro-inflammatory cytokines, such as interleukin-6 (IL-6), interleukin-1β (IL-1β), tumor necrosis factor-α (TNF-α), and interleukin-18 (IL-18), while reducing anti-inflammatory cytokines like IL-10 and IL-13 (Kang et al., 2016; Yi Ye et al., 2021; Yi X. et al., 2021). Dysregulated cytokine levels impair synaptic plasticity and worsen depressive symptoms (Kim et al., 2017). In addition, reduced oxygen and ATP concentrations in brain tissues further impair neuronal function, increasing the vulnerability to depression (Che et al., 2021).

In animal models of PSD, Wu et al. demonstrated that stroke combined with chronic stress elevated IL-18 levels, promoting depressive-like behaviors through the IL-18 receptor/NKCC1 signaling pathway (Wu et al., 2020). Other studies have identified elevated levels of growth differentiation factor-15 (GDF-15) as a biomarker for PSD. Lu et al. found that GDF-15 levels were over twice as high in PSD patients compared to non-depressed stroke patients, and Zang et al. reported that GDF-15 was independently associated with PSD (Lu X. et al., 2020; Zang et al., 2022). Additional biomarkers, including homocysteine (Hcy) and high-sensitivity C-reactive protein (Hs-CRP), have also been linked to increased PSD risk, suggesting that chronic inflammation is closely tied to its pathogenesis (Tang et al., 2016; Cheng et al., 2018). TCM interventions modulate neuroinflammatory responses. Poria cocos inhibits the NLRP3 inflammasome, reducing pro-inflammatory cytokine production and restoring immune balance. *Salvia miltiorrhiza* Bunge (Lamiaceae; Dan Shen) suppresses IL-6 production, alleviating depressive symptoms and promoting emotional stability (Bian et al., 2023).

## 4 Gut-brain axis and PSD: a complex network of interactions

The enteric nervous system (ENS), forming part of the gut-brain axis, is a vast network of neurons within the gastrointestinal tract. It enables bidirectional communication between gut microbiota and the brain through neuroendocrine, immune, and metabolic pathways, thus influencing emotional regulation, cognition, and systemic health (Begum et al., 2022). Gut microorganisms, including bacteria and fungi, play a pivotal role in fermenting undigested food to produce essential energy sources and metabolites that support immune and digestive functions. The gut microbiota communicates with the brain via the ENS and vagus nerve, impacting central nervous system (CNS) processes, including mood and behavior (Han et al., 2022).

### 4.1 Gut dysbiosis and neurotransmitter imbalance in PSD

Dysbiosis, or the imbalance of gut microbial populations, has been strongly associated with PSD. Stroke survivors with PSD often exhibit reduced microbial diversity, marked by an increase in pathogenic bacteria and a decrease in anti-inflammatory species (Liang et al., 2015). This imbalance interferes with neurotransmitter metabolism, particularly serotonin (5-HT) and norepinephrine (NE), contributing to depressive symptoms (Jiang et al., 2021). Experimental studies have shown that transplanting gut microbiota from PSD patients into healthy rodents induces depressive behaviors, such as weight loss, decreased activity, and anhedonia (Frank et al., 2019). Additionally, gut dysbiosis may disrupt the synthesis of essential cofactors, such as vitamin B12 and folic acid, critical for homocysteine metabolism. Elevated homocysteine levels, commonly observed in PSD patients, impair monoamine neurotransmitter synthesis, contributing to depressive symptoms (Geng et al., 2017; Hu S. et al., 2019)

### 4.2 HPA axis dysregulation and the gut-brain axis in PSD

The hypothalamic-pituitary-adrenal (HPA) axis is closely linked to the gut-brain axis. Dysbiosis affects the HPA axis by altering microbial metabolites, which influence the release of corticotropin-releasing hormone (CRH). In stressful situations, activation of the HPA axis leads to increased cortisol levels, which impair gut barrier function, disrupt microbial balance, and exacerbate mood disturbances (Zhanina et al., 2022). PSD patients often exhibit elevated cortisol levels, underscoring the contribution of HPA axis dysregulation to the development of depressive symptoms (Yang et al., 2021). The overlapping mechanisms—reduced neurotransmitter synthesis and HPA axis dysregulation—highlight the importance of maintaining a balanced gut-brain axis for effective PSD management.

### 4.3 Neuroinflammation and the role of gut dysbiosis in PSD

Chronic neuroinflammation is a hallmark of PSD, often driven by microbial by-products such as lipopolysaccharides (LPS) entering circulation through a compromised intestinal barrier (Maes et al., 2012). Elevated levels of pro-inflammatory cytokines—such as interleukin-6 (IL-6), interleukin-1β (IL-1β), and tumor necrosis factor-α (TNF-α)—have been reported in PSD patients, along with a reduction in brain-derived neurotrophic factor (BDNF) (Rao et al., 2021). Gut dysbiosis also contributes to blood-brain barrier (BBB) dysfunction, allowing neurotoxins to reach the brain, further aggravating depressive symptoms.

### 4.4 TCM interventions targeting the gut-brain axis in PSD

Given the intricate relationship between the gut-brain axis and PSD, TCM offers promising therapeutic strategies. TCM formulations such as Chaihu Shugan Powder (CSP) promote the growth of beneficial gut bacteria and reduce pro-inflammatory species, alleviating depressive symptoms (Liu et al., 2021). *Salvia miltiorrhiza* Bunge (Lamiaceae; Dan Shen) has been shown to modulate the PI3K-AKT pathway and enhance vagus nerve signaling, improving gut-brain axis communication and emotional regulation (Bian et al., 2023).

Furthermore, combining probiotics with TCM formulations has yielded promising results by enhancing anti-inflammatory cytokine production and reducing serum cortisol levels, leading to improved mood and reduced neuroinflammation (Rao et al., 2021). This integrative approach demonstrates the potential of personalized medicine strategies that target e gut-brain axis to treat PSD.

## 5 Herbal interventions for enhancing recovery in PSD

The theories of Chinese medicine emphasize individualized diagnosis and treatment according to each patient and their environment. The main methods of treating PSD include invigorating blood circulation and removing blood stasis, detoxifying the liver and relieving depression, invigorating the spleen and strengthening qi, and tonifying the kidneys and essence. Invigorating blood circulation and removing blood stasis aim to improve qi and blood circulation by dredging meridians and collaterals, using medicines such as *Ligusticum chuanxiong* S.H.Qiu, Y.Q.Zeng, K.Y.Pan, Y.C.Tang & J.M.Xu (Apiaceae; Chuan Xiong), *S. miltiorrhiza* Bunge (Lamiaceae; Dan Shen), and *Paeonia lactiflora* Pall. (Paeoniaceae; Shao Yao). Detoxifying the liver and relieving depression focus on regulating the liver, qi, and calming the mind, with herbs like *Bupleurum chinense* DC. (Apiaceae; Bei Chai Hu), *Cyperus rotundus* L. (Cyperaceae; Xiang Fu), and *Curcuma aromatica* Salisb. (Zingiberaceae; Yu Jin). Strengthening the spleen and vital energy is particularly for patients with deficiency of the heart and spleen, employing medicines *G. uralensis* Fisch. ex DC. (Fabaceae; Gan Cao), *A. membranaceus* Fisch. ex Bunge (Fabaceae; Huang Qi), *Fructus aurantii* (Rutaceae; Zhi Shi), *Poria cocos* (Schw.) Wolf (Polyporaceae; Fu Ling). The kidney tonic drugs are for patients with deficiency of spleen and kidney, such as the use of *Rehmannia glutinosa* (Gaertn.) Libosch. ex DC. (Orobanchaceae; Di Huang)*, Cornus officinalis* Siebold & Zucc. (Cornaceae; Shan Zhu Yu)*, Lycium barbarum* L. (Solanaceae; Goji Berry) *and Morinda officinalis* F.C.How (Rubiaceae; Ba Ji Tian). By regulating the internal organs with these herbal treatments, the functions of the liver, heart, spleen, and kidneys are restored on an individual basis, achieving balance and coordination among the internal organs (Table 2).

**TABLE 2:** Pharmacological properties and potential mechanisms of classic herbs for treating PSD.

Herb	Latin	Meridians	Therapeutic properties	Major extracts	Modern pharmacological effects	Key findings
Chuanxiong	Ligusticum chuanxiong S.H.Qiu, Y.Q.Zeng, K.Y.Pan, Y.C.Tang & J.M.Xu	Liver, Gallbladder, Pericardium.	Move blood, Relieve pain, Expel wind.	Alkaloids, Volatile oils, organic acids.	Anti-inflammatory, Antioxidant, Antitumor.	1) chuanxiongzine A upregulates the cAMP-CREB-BDNF pathway and increasing BDNF expression (Yu et al., 2015).
						2) Ligustilide upregulates NE and DA content in hippocampus (Wu et al., 2019).
Dan Shen	Salvia miltiorrhiza Bunge	Heart, Liver.	Invigorate blood, Clear heart, Sooth liver	Tanshinones, Salvianolic acids, flavonoids	Neuroprotective, Anti-inflammatory, Antioxidant	1) Tanshinone IIA can regulate the ERK-CREB-BDNF pathway to fight depression (Lu et al., 2020a).
						2) CPT can regulate the PI3K-AKT pathway and exert antidepressant effects (Bian et al., 2023).
White Peony Root	Paeonia lactiflora Pall	Liver, Spleen.	Nourish blood, Regulate liver	Monoterpene glycosides, Triterpenes, Flavonoids	Neuroprotective, Anti-inflammatory, Antioxidant	1) PT can increase the expression levels of BDNF and CREB proteins in the hippocampus of PSD rats, providing neuroprotective and antidepressant effects (Hu et al., 2019a).
Red Peony Root	Paeonia veitchii Lynch	Liver, Spleen.	Cool blood, Dispel blood stasis		Anti-inflammatory, Neuroprotective	
Chai Hu	Bupleurum chinense	Liver, Gallbladder	Soothe liver, Relieve depression, Clear heat	Saikosaponins	Antipyretic, Antidepressant, Anti-inflammatory	1) SSA can improve depressive-like behaviors through the p-CREB/BDNF pathway (Wang et al., 2021a).
Xiang Fu	Cyperus rotundus L.	Liver, Spleen, Triple burner	Regulates Qi, Relieve pain	Volatile oils, Flavonoids, Triterpenoids, Alkaloids	Antidepressant, Anxiolytic, Anti-inflammatory	1) Cyperus rotundus L can improve the depressive state in mice by inhibiting the expression of 5-HT and MAO-A (Wang et al., 2013).
Yu Jin	Curcuma aromatica Salisb	Heart, Liver, Gallbladder	Promote blood circulation, Relieve depression	Terpenes, Curcuminoid.	Neuroprotective, Antidepressant, Anti-inflammatory	1) Curcuma wenyujin extract can promote angiogenesis in the CA3 region of the hippocampus by increasing the expression of VEGF and its receptor FLK-1 to exert an antidepressant effect (Zhao et al., 2011; Qian et al., 2012).
Gan Cao	Glycyrrhiza uralensis Fisch. ex DC.	Heart, Lung, Spleen, Stomach	Tonify Qi, Moisten lung, Relieve cough	Flavonoids, Saponins, Polysaccharides, Coumarins	Antiviral, Anti-inflammatory, Immunomodulatory	1) Licorice glycosides can upregulate the expression of Bcl-2 protein and downregulate Bax apoptotic protein to provide neuronal protection (Wang et al., 2021).
Huang Qi	Astragalus membranaceus Fisch. ex Bunge	Spleen, Lung	Tonify Qi, Strengthen spleen, Promote urination	Astragalosides, Astragalus polysaccharides, Flavonoids	Neuroprotective, Antioxidant, Immunomodulatory	1) AsVI can upregulate the NRG1-mediated MEK/ERK pathway and improve depressive-like behavior (Chen et al., 2022).
Zhi Shi	Fructus aurantii	Spleen, Stomach	Regulate Qi, Relieve distension	Flavonoids, Coumarins, Alkaloids, and Volatile oils	Antidepressant, Neuroprotective, Antioxidant	1) Naringenin and hesperetin can modulate the serotonin, norepinephrine, and dopamine systems to exert antidepressant effects (Yi et al., 2010; Yi et al., 2011).
Fu Ling	Poria cocos (Schw.) Wolf	Heart, Spleen, Lung, Kidney	Drain dampness, Strengthen spleen	Triterpenes, Polysaccharides	Antidepressant, Anti-inflammatory, Antioxidant	1) PCW can exert anti-inflammatory and antidepressant effects by reducing the DA and 5-HT in rats and the markers p38, NF-κB, and TNF-α (Huang et al., 2020)
						2) TTWC can exert antidepressant effects by regulating neurotransmitters, HPA axis and NLRP3 signaling pathway Pan et al., 2022.
Shu Di Huang	Rehmannia glutinosa (Gaertn.) Libosch. ex DC.	Liver, Kidney	Tonify blood, Nourish Yin	Catalpol, Rehmannioside, Polysaccharides	Neuroprotective, Anti-inflammatory, Antidepressant	1) Catalpol can exert multiple antidepressant effects by upregulating the PI3K/Akt/Nrf2/HO-1 pathway, downregulating the ERK1/2/Nrf2/HO-1 pathway, and regulating the TrkB signaling pathway (Wang et al., 2021b; Wu et al., 2022; Wu et al., 2024).
						2) RGP can inhibit oxidative stress and protect neurons by regulating the AKT/mTOR pathway (Yang et al., 2024a).
Shan Zhu Yu	Cornus officinalis Siebold & Zucc.	Liver, Kidney	Tonify liver and kidneys, Secure essence	Ridoid glycosides, Tannins, Triterpenes, Organic acids, Flavonoids	Neuroprotective, Antidepressant, Anti-inflammatory	1) Morroniside can regulate the MiR-409-3p-mediated BDNF/TrkB signaling pathway to inhibit neuronal apoptosis (Qian et al., 2024).
						2) Loganin can exert antidepressant effects by activating the BDNF-TrkB signaling pathway (Gong et al., 2023).
Goji Berry	Lycium barbarum L.	Liver, Kidney	Tonify liver and kidneys, Nourish blood	Polysaccharides, Betaine, Carotenoids, Flavonoids	Neuroprotective, Antioxidant, Antidepressant	1) LBP can regulate Nrf2/HO-1 and thus reduce oxidative stress and mitochondrial damage (Yang et al., 2023).
						2) LbGp can provide neuroprotection by downregulating ferroptosis-related factors in the medial prefrontal cortex (Zhao et al., 2021b; Dai et al., 2023).
Ba Ji Tian	Morinda officinalis F.C.How	Liver, Kidney	Tonify kidney Yang, Strengthen bones	Anthraquinones, Iridoids, and Polysaccharides	Antidepressant, Neuroprotective, Anti-inflammatory	1) MOOs can exert anti-inflammatory and antidepressant effects by regulating the IκB/NF-κB p65 pathway and thus downregulating the NLRP3 inflammasome (Li et al., 2021).

### 5.1 Activating blood circulation and removing blood stasis herbs

*Ligusticum chuanxiong* S.H.Qiu, Y.Q.Zeng, K.Y.Pan, Y.C.Tang & J.M.Xu (Apiaceae; Chuan Xiong): Contains alkaloids and volatile oils that activate the cAMP-CREB-BDNF pathway, increasing NE and DA, improving synaptic plasticity and mood stability (Wu et al., 2019; Yu et al., 2022).

*Salvia miltiorrhiza* Bunge (Lamiaceae; Dan Shen): Offers neuroprotection through tanshinones and salvianolic acids. Tanshinone IIA activates the ERK-CREB-BDNF pathway to a lleviate depression (Lu J. et al., 2020). Sodium tanshinone IIA sulfonate enhances function in ischemic stroke models (Wang Z. et al., 2022). and cryptotanshinone regulates the PI3K-AKT pathway and gut microbiota (Bian et al., 2023).

*Paeonia lactiflora* Pall. (Paeoniaceae; Shao Yao) and *Paeonia veitchii* Lynch (Paeoniaceae; Chuan Chi Shao): Known for anti-inflammatory effects, these herbs modulate neurotransmitter levels, reducing oxidative stress. Paeoniflorin boosts BDNF, enhancing synaptic plasticity and cognitive function (Hu M. Z. et al., 2019; Wang X. L. et al., 2021).

### 5.2 Relieving liver disease and dispel depression herbs

*Bupleurum chinense* DC. (Apiaceae; Chai Hu): Saikosaponins increase serotonin and dopamine via the p-CREB/BDNF pathway (Wang A. R. et al., 2021). Ping et al. reported improved pharmacokinetics and enhanced antidepressant effects when saikosaponin A (SSA) was combined with paeoniflorin, suggesting a synergistic action. Additionally, other components such as saikosaponin D (SSD), quercetin, bupleurum polysaccharides, kaempferol, and baicalin have demonstrated antidepressant properties (Yin et al., 2023).

Cyperus *rotundus* L (Cyperaceae; Xiang Fu): balances qi and soothes the liver, essential in TCM for regulating emotions. Its extracts have been shown to improve depressive symptoms by increasing 5-HT levels and inhibiting monoamine oxidase A (MAO-A) activity (Lu et al., 2022; Wang F. et al., 2022).

Curcuma *aromatica* Salisb (Zingiberaceae; Yu Jin): Curcumin, its primary bioactive compound, has exhibited significant antidepressant effects through behavioral models, including the tail suspension test. Furthermore, curcumin promotes hippocampal angiogenesis by upregulating vascular endothelial growth factor (VEGF) and FLK-1 expression, thereby improving cognitive function and mood stability (Zhao et al., 2011; Qian et al., 2012).

### 5.3 Strengthen the spleen and benefit the qi herbs

*Glycyrrhiza uralensis* Fisch. ex DC (Fabaceae; Gan Cao): contains glycyrrhizic acid, which exhibits anti-inflammatory properties. It promotes neuronal survival by enhancing Bcl-2 expression, reducing neuroinflammation, and alleviating depressive symptoms (Wang et al., 2023; Wang et al., 2021).

*Astragalus membranaceus* Fisch. ex Bunge (Fabaceae; Huang Qi): activates the EGFR/MAPK pathway, promoting neuronal recovery and emotional stability. modulates the gut-brain axis, and supports mood and cognitive function in PSD patients (Chen et al., 2022).

*Fructus aurantii* (Rutaceae; Zhi Ke): exerts antidepressant effects through its flavonoid content, such as naringenin and hesperetin, which regulate dopamine receptor activity. These active compounds contribute to emotional wellbeing by restoring neurotransmitter balance, enhancing mood, and supporting cognitive function (Yi et al., 2010; Yi et al., 2011).

*Poria cocos* (Schw.) Wolf (Polyporaceae; Fu Ling): inhibits the NLRP3 inflammasome, reducing depressive behaviors and inflammatory markers (Huang et al., 2020). Additionally, the total triterpenes of *Poria cocos* have been shown to exhibit antidepressant effects through modulation of neurotransmitter pathways, further validating its role in PSD management (Pan et al., 2022).

### 5.4 Invigorating the kidneys and benefit the vital essence herbs

Rehmannia *glutinosa* (Gaertn.) Libosch. ex DC. (Orobanchaceae; Shu Di Huang): mitigates oxidative stress via the PI3K/Akt/Nrf2 pathway, with catalpol enhancing synaptic plasticity and neurogenesis through the TrkB pathway (Bhattamisra et al., 2019; Song et al., 2021; Sun et al., 2021; Wang Y. L. et al., 2021). *Rehmannia glutinosa* polysaccharides (RGP) further mitigate oxidative stress and promote autophagy, providing neuroprotection in PSD models (Yang Y. et al., 2024; Wang J. et al., 2021; Wu et al., 2022; Wu et al., 2024).

Cornus *officinalis* Siebold & Zucc. (Cornaceae; Shan Zhu Yu): alleviates depressive-like symptoms by activating the BDNF/TrkB signaling pathway. Morroniside has been shown to reduce PSD-related symptoms by improving synaptic function and enhancing hippocampal plasticity through miRNA modulation (Qian et al., 2024). Additionally, logani exhibits neuroprotective and anti-inflammatory properties, further contributing to mood stabilization (Gong et al., 2023).

Lycium *barbarum* L. (Solanaceae; Goji Berry), linked to liver and kidney meridians, is traditionally used to address fatigue and yin deficiency. Its polysaccharides (LBP), constituting a major portion of its active compounds, have demonstrated antidepressant effects by reducing oxidative stress through the Nrf2/HO-1 pathway and mitigating anxiety-like behaviors (Zhou et al., 2021; Dai et al., 2023; Yang et al., 2023; Zhao F. et al., 2021).

*Morinda officinalis* F.C.How (Rubiaceae; Ba Ji Tian): is known for treating kidney yang deficiency and rheumatic pain. Morinda officinalis oligosaccharides (MOOs), inhibit NLRP3 inflammasome activation, reducing neuroinflammation and alleviating depressive behaviors in PSD models (Li et al., 2021). This dual anti-inflammatory and neuroprotective effect underscores its therapeutic relevance in managing PSD.

## 6 Classical formulations and Chinese patent medicines

**FIGURE 3:**
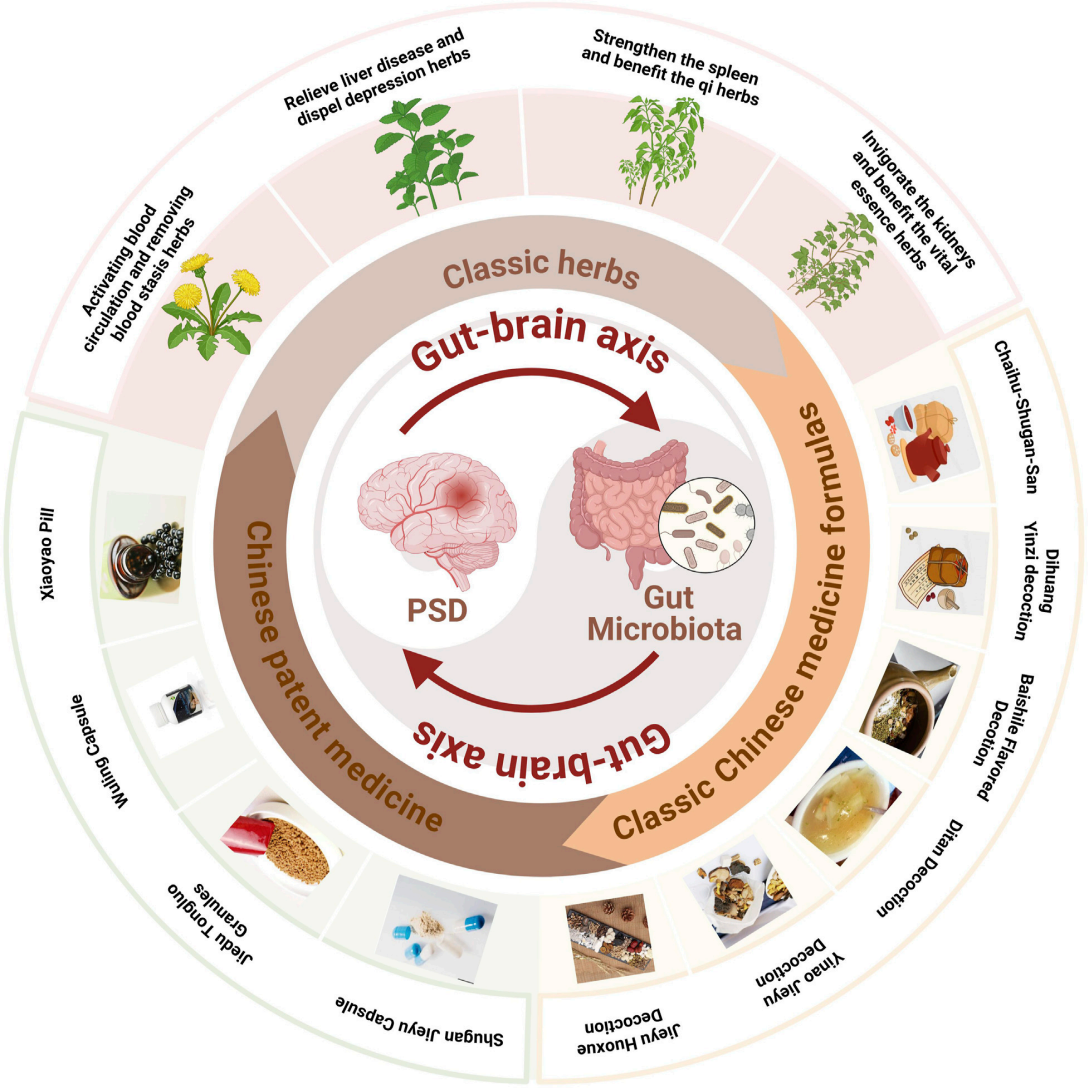
TCM regulates the gut-brain axis to treat PSD.

TCM formulas, composed of multiple herbs, offer a more comprehensive approach to managing PSD by addressing both emotional and physiological imbalance (Table 3). In addition, the close connections and related potential mechanisms among herbal medicines, classic Chinese medicine formulas and Chinese patent medicines for the treatment of stroke depression have been presented in Figure 3.

**TABLE 3:** Modern pharmacologic mechanisms and clinical studies of classical formulas and proprietary Chinese medicines for the treatment of PSD.

Category	Name	Constituent/ Active components	Pathological Model	Modern Pharmacology	Clinical Application
Classic Chinese Medicine Formulas	Chaihu-Shugan-San (CHSG)	Bupleurum, Cyperus, Ligusticum chuanxiong, Citrus, Fructus Aurantii, White Peony, Licorice.	MCAO+CUMS rats	Increase NE, 5-HT, DA and BDNF expression (Jia et al., 2023).	1) Combined TES treatment of PSD patients improves 5-HT and BDNF levels in the brain (Li and Liu, 2022).
			MCAO+CUMS rats	Increase BDNF and TrkB expression and reduces inflammatory factors IL-6 and TNF-α (Hu et al., 2020).	2) Combine Western medicines including citalopram hydrobromide, haloperidol melittin and fluoxetine can relieve depressive symptoms and significantly reduce the adverse effects of single medication (Huang and Zeng, 2015; Hu et al., 2018; Wang et al., 2019).
			MCAO+CUMS rats	Regulate the JAK/STAT3-GSK3β/PTEN/Akt pathway and microglial polarization alleviates neuroinflammation (Fan et al., 2023a).	
	Dihuang Yinzi Decoction (DHYZ)	Radix Rehmanniae Praeparata, Cornu Cervi Pantotrichum, Dendrobium nobile, Cistanche deserticola, Bacopa monnieri	MCAO+CUMS rats	Inhibit ferroptosis through P53/SLC7A11/GPX4 pathway to provide neuroprotection and relieve depression (Yang et al., 2024b).	DHYZ is beneficial for neurological rehabilitation and prognosis in patients with PSD (Yu et al., 2015).
	Baishile Flavored Decotion (MBD)	Turmeric, Forsythia penetrans, Ginseng.	MCAO+CUMS rats	Reduce IL-1β and NPY in serum and intestinal tissues and modulates the P2X7R/NLRP3 signaling pathway to exert antidepressant effects (Liu et al., 2022).	MBD can modulate brain gut peptides to reducing inflammatory responses and provide neuroprotection (Yuan et al., 2024).
	Ditan Decoction	Semen Xie, Poria and Calamus.	MCAO+ lipiodol injection (PSD model) rats	Reduce the depressive symptoms in PSD rats by regulating the expression of GAS, NPY, and CGRP (Zhang et al., 2020).	Ditan decoction inhibit the levels of NF-κB and miR-146a within the serum and improve neurological function scores in PSD patients after stroke (Luo et al., 2019b).
	Yinao Jieyu Decoction	Acanthopanax Senticosus, Curcuma aromatica, Paeonia lactiflora Pall, Gardenia jasminoides.	CUMS rats	inhibit NLRP3 activation in rat hippocampus and prefrontal cortex tissues to alleviate depression-like symptoms (Zhang et al., 2023b).	Combine acupuncture can reduce the incidence of treatment adverse effects, improve serum total bilirubin levels and improve patients' depression (Du et al., 2021).
	Jieyu Huoxue Decoction	Citrus aurantium dulcis, Acanthopanax spinosa, Salvia miltiorrhiza, Aromatica odorata, Paeonia lactiflora Pall.	PSD patients	Reduce serum BDNF, NGF, DA, 5-HT and NE levels and levels of amino acid neurotransmitters Glu, Asp, Gly, GABA thus providing antidepressant effects (Wu et al., 2021).	Combine Western medicines including fluoxetine, paroxetine hydrochloride and acupuncture can reduce adverse effects and improve the psychological status of PSD patients (Chen et al., 2019; Ren et al., 2023).
Chinese Patent Medicine	Shugan Jieyu Capsule (SG)	Hypericum perforatum and Acanthopanax, etc.	CUMS rats	Hypericin can increase the brain level of BDNF, inhibit glutamate release, reduce Ca2+ influx and activate BDNF-TrkB-PI3K/Akt pathway to alleviate depressive-like behaviors (Chang and Wang, 2010).	1) SG can regulate the levels of 5-HT and NE in PSD patients and inhibit the level of inflammation, thereby alleviating depression (Liu et al., 2019).
			CUMS mice	Eleutheroside can inhibit the inflammatory response and provide neuroprotection by regulating the HPA axis and increasing the levels of DA and GABA (Bian et al., 2018; Qi et al., 2020).	2) Combine Western medicines including fluoxetine, deanxit, escitalopram, sertraline and tandospirone Citrate to improve depressive behaviors in PSD patients (Gu et al., 2018; Ye et al., 2021; Jiang, 2023; Shu, 2023).
	Jiedu Tongluo Granules (JDTLG)	Ginseng, Ginkgo biloba, Gardenia and Chuanxiong.	MCAO+CUMS rats	Modulate NMDAR/BDNF pathway, reduce Glu levels and increase GABA levels to provide neuroprotection (Song et al., 2015).	JDTLG may have antidepressant and neuroprotective effects by modulating the liver (Zhao et al., 2021a).
	Wuling Capsule	Wuling mycelia powder and Epimedium, etc.	PSD patients	Regulate the expression of neurotransmitters and neurocellular factors and the PI3K/Akt signaling pathway to improve depressive behaviors (Zheng et al., 2024).	Combine Western medicines including oryzanol, xylariasp, flupentixol and escitalopram oxalate to improve depression and sleep in PSD patients (Shi, 2021; Tian et al., 2021; Zhao et al., 2023).
	Xiaoyao Pill	Chaihu, Angelica sinensis and Atractylodes macrocephala, etc.	PSD patients	Reduce 5-HT concentrations within the serum to provide an antidepressant therapeutic effect (Hu et al., 2024).	Combine Western medicines including fluoxetine, flupentixol and escitalopram oxalate to improve depressive symptoms and promotes recovery of neurological function in PSD patients (Wang and Ni, 2014; Zeng et al., 2018; Hu et al., 2024).

### 6.1 Chaihu Shugan Powder (CSP)

This classical TCM formula, documented in the Ming Dynasty’s Jingyue Quanshu, has been used for nearly four centuries to treat emotional disorders, particularly those associated with liver qi stagnation and depression. CSP consists of seven core herbs: *Bupleurum chinense* DC. (Apiaceae; Chai Hu), *C. rotundus* L. (Cyperaceae; Xiang Fu), *L. chuanxiong* S.H.Qiu, Y.Q.Zeng, K.Y.Pan, Y.C.Tang & J.M.Xu (Apiaceae; Chuan Xiong), *Citrus reticulata* Blanco (Rutaceae; Chen Pi), *Citrus aurantium* L (Rutaceae; Zhi Ke), *P. lactiflora* Pall. (Paeoniaceae; Bai Shao), and *G. uralensis* Fisch. ex DC. (Fabaceae; Gan Cao). These herbs work synergistically to relieve lumbar pain, regulate qi, and alleviate emotional distress. Recent pharmacological studies show that CSP significantly enhances monoamine neurotransmitter levels in PSD patients, promoting emotional stabilization (Liu et al., 2020; Gao et al., 2022). Furthermore, CSP has been shown to reduce neuroinflammation by lowering serum TNF-α levels and hippocampal NF-κB expression, with higher dosages correlating with stronger anti-inflammatory effects (Fan Q. et al., 2023). Gao et al. further demonstrated reductions in IL-6 and TNF-α, confirming the anti-inflammatory potential of this formula (Gao et al., 2021; Hu et al., 2020; Jia et al., 2023).

In clinical practice, CSP has proven effective when combined with western antidepressants such as citalopram and haloperidol, enhancing therapeutic outcomes while reducing adverse side effects (Kwon et al., 2019; Hu et al., 2018; Huang and Zeng, 2015; Li et al., 2022; Wang et al., 2019) This integration of TCM with conventional medicine highlights CSP’s potential as an adjunct treatment for PSD, offering both mood stabilization and neuroprotection.

### 6.2 Dihuang Yinzi decoction (DHYZ)

First documented in the Xuan Ming Lun Fang, DHYZ consists of twelve herbs, including *R. glutinosa* (Gaertn.) Libosch. ex DC. *(prepared root)* (Orobanchaceae; Di Huang), *Gynochthodes officinalis* (F.C.How) Razafim. & B.Bremer (Rubiaceae; Ba Ji Tian), *C. officinalis* Siebold & Zucc. (Cornaceae; Shan Zhu Yu), *Dendrobium nobile* Lindl. (Orchidaceae; Shi Hu), *Cistanche deserticola* Ma (Orobanchaceae; Rou Cong Rong), *Aconitum carmichaelii* Debx. (Ranunculaceae; Fu Zi), *Schisandra chinensis* (Turcz.) Baill. (Schisandraceae; Wu Wei Zi), *Cinnamomum cassia* Nees (Lauraceae; Guan Gui), *Wolfiporia extensa* (Peck) E. Horak. (Polyporaceae; Fu Ling), *Ophiopogon japonicus* (Thunb.) Ker Gawl. (Asparagaceae; Mai Dong), *Acorus gramineus* Aiton (Acoraceae; Shi Chang Pu), and *Polygala tenuifolia* Willd. (Polygalaceae; Yuan Zhi). Traditionally used to treat neurological disorders, DHYZ has shown effectiveness in rodent models by reducing apoptosis and enhancing memory (Yu et al., 2015; An et al., 2017). Researchers found that DHYZ alleviates PSD symptoms by inhibiting ferroptosis through the P53/SLC7A11/GPX4 pathway, providing neuroprotection (Yang Z. et al., 2024).

Other classical formulas, such as Baishile Flavored Decotion (MBD) (Liu et al., 2022), Ditan Decoction (Zhang et al., 2020), Yinao Jieyu Decoction (Zhang X. et al., 2023) and Jieyu Huoxue Decoction (Wu et al., 2021), have also demonstrated effectiveness in managing PSD, either as standalone treatments or combined with Western therapies.

### 6.3 Shugan Jieyu Capsule (SG)

Approved by the China National Medical Products Administration in 2008, SG is the first herbal product specifically indicated for depression. Its key components are *Hypericum perforatum* L. (Hypericaceae; Guan Ye Jin Si Tao) and *Acanthopanax* (Decne. & Planch.) Witte (Araliaceae; Ci Wu Jia), which exhibit calming, cognitive-enhancing, and anti-inflammatory properties. Hypericin modulates the HPA axis, inhibits glutamate release, and boosts BDNF expression (Chang and Wang, 2010). Quercetin activates the BDNF-TrkB-PI3K/Akt pathway, further alleviating depressive symptoms (Qi et al., 2020). Acanthopanax’s active compounds, eleutherosides B and E, reduce depressive behaviors, while syringin increases dopamine and GABA levels (Bian et al., 2018). Additional components, such as emodin and syringaresinol, also exhibit antidepressant properties (Bonaterra et al., 2020; Zhang et al., 2021). Clinical studies show that SG increases norepinephrine and serotonin levels, with enhanced outcomes when combined with fluoxetine (Yao et al., 2020; Jiang et al., 2023; Liu et al., 2019; Shu et al., 2018).

### 6.4 Jiedu Tongluo Granules (JDTLG)

A proprietary TCM formulation, contains *Panax ginseng* C.A.Mey. (Araliaceae; Ren Shen)*, Scutellaria baicalensis* Georgi (Lamiaceae; Huang Qin), *Ginkgo biloba* L. (Ginkgoaceae; Yin Xing Ye)*, H. perforatum* L. (Hypericaceae; Guan Ye Lian Qiao)*, Gardenia* J.Ellis (Rubiaceae; Zhi Zi Hua)*, Gastrodia elata Blume* (Orchidaceae; Tian Ma), and *L. chuanxiong* S.H.Qiu, Y.Q.Zeng, K.Y.Pan, Y.C.Tang & J.M.Xu (Apiaceae; Chuan Xiong). It enhances physical recovery and alleviates depressive symptoms in PSD patients (Song et al., 2015). Zhao et al. demonstrated that JDTLG exerts neuroprotective effects by modulating the NMDAR/BDNF pathway, lowering glutamate levels, and increasing GABA concentrations, stabilizing mood (Zhao A. et al., 2021).

Additionally, other compound Chinese medicines, such as Wuling Capsule (Zheng et al., 2024) and Xiaoyao Pills (Hu et al., 2024) have been shown to improve depressive behaviors, either alone or in combination with Western pharmacotherapies.

### 6.5 Baishile flavored decoction

Baishile Flavored Decoction, containing *Curcuma longa* L. (Zingiberaceae; Jiang Huang), *Forsythia suspensa* (Thunb.) Vahl (Oleaceae; Lian Qiao), and *Panax ginseng* C.A.Mey. (Araliaceae; Ren Shen), exerts antidepressant effects primarily through modulation of the P2X7R/NLRP3 signaling pathway. Studies in MCAO + CUMS rat models have shown that Baishile significantly reduces IL-1β and neuropeptide Y (NPY) levels in serum and intestinal tissues, leading to reduced neuroinflammation and improved neurological function (Liu et al., 2022). Moreover, Clinical studies have demonstrated the ability of MBD to exert neuroprotective effects and reduce inflammatory responses by modulating brain-gut peptides (Yuan et al., 2024).

### 6.6 Ditan decoction

Ditan Decoction, composed of *Pinellia ternata* (Thunb.) Bremer (Araceae; Ban Xia), *Poria cocos* (Schw.) Wolf (Polyporaceae; Fu Ling), *Arisaema cum bile* L. (Araceae; Tan Nan Xing), *Acorus calamus* L. (Acoraceae; Shi Chang Pu), *Citri Grandis Exocarpium* (Rutaceae; Ju Hong), *Poncirus trifoliata* (L.) Raf. (Rutaceae; Zhi Shi), *Bambusae Caulis In Taenias* (Poaceae; Zhu Ru), *Panax ginseng* C.A.Mey. (Araliaceae; Ren Shen) and *G. uralensis* Fisch. ex DC. (Fabaceae; Gan Cao) has been found to regulate key neurotransmitters, including GAS, NPY, and CGRP, thereby alleviating depression in PSD rat models (Zhang et al., 2020). Clinical studies further indicate that Ditan Decoction inhibits NF-κB and miR-146a expression in serum, which correlates with reduced neuroinflammatory responses and improved neurological function scores in PSD patient post-stroke (Luo W. et al., 2019).

### 6.7 Yinao jieyu decoction

Yinao Jieyu Decoction, containing *Acanthopanax senticosus* (Rupr. et Maxim.) Harms (Araliaceae; Ci Wu Jia), *C. aromatica* Salisb. (Zingiberaceae; Yu Jin), *S. chinensis* (Turcz.) Baill. (Schisandraceae; Wu Wei Zi) and *Gardenia jasminoides* J.Ellis (Rubiaceae; Zhi Zi Hua), has been reported to alleviate depressive-like symptoms in CUMS rat models via NLRP3 inflammasome inhibition in hippocampal and prefrontal cortex tissues (Zhang S. et al., 2023). Additionally, when combined with acupuncture, Yinao Jieyu Decoction has been observed to reduce the incidence of adverse effects, improve serum bilirubin levels, and enhance PSD recovery (Du et al., 2021).

### 6.8 Jieyu huoxue decoction

Jieyu Huoxue Decoction, formulated with *P. trifoliata* (L.) Raf (Rutaceae; Zhi Shi), *Acanthopanax spinosa* (L.). Siebold & Zuccarin (Araliaceae; Ci Wu Jia), *S. miltiorrhiza* Bunge (Lamiaceae; Dan Shen), *C. rotundus* L. (Cyperaceae; Xiang Fu), *Paeoniae Radix Alba* (Paeoniaceae; Bai Shao), *Bupleurum chinense* DC. (Apiaceae; Chai Hu) and *Angelica sinensis* (Oliv.) Diels (Apiaceae; Dang Gui), exerts antidepressant effects by regulating monoamine neurotransmitters (BDNF, NGF, DA, 5-HT, and NE) and amino acid neurotransmitters (Glu, Asp, Gly, and GABA) (Wu et al., 2021).Clinical data suggest that combining Jieyu Huoxue Decoction with Western antidepressants, such as fluoxetine and paroxetine hydrochloride, as well as acupuncture, enhances psychological recovery and reduces medication side effects in PSD patients (Chen et al., 2019; Ren et al., 2023).

### 6.9 Wuling capsule

Wuling Capsule, derived from *Wuling Mycelia Powder*, acts via the PI3K/Akt signaling pathway, which is crucial for neuroprotection and synaptic plasticity. Studies in PSD patients have confirmed that Wuling Capsule modulates neurotransmitter expression and enhances neurocellular factor activity, leading to improved depressive symptoms and sleep quality (Zheng et al., 2024; Shi, 2021; Tian et al., 2021; Zhao et al., 2023).

### 6.10 Xiaoyao pills

Xiaoyao Pills containing *Bupleurum chinense* DC. (Apiaceae; Chai Hu), *A. sinensis* (Oliv.) Diels (Apiaceae; Dang Gui), *Paeoniae Radix Alba* (Paeoniaceae; Bai Shao), *Atractylodes macrocephala* Koidz. (Asteraceae; Bai Zhu), *W. extensa* (Peck) E. Horak. (Polyporaceae; Fu Ling), *Mentha canadensis* L. (Lamiaceae; Bo he), *Zingiber officinale* Roscoe (Zingiberaceae; Sheng Jiang) and *Glycyrrhizae radix et rhizoma praeparata* (Fabaceae; Mi Zhi Gan Cao) is widely used in PSD patients due to its ability to modulate 5-HT levels in serum, directly impacting mood regulation (Hu et al., 2024; Wang et al., 2013; Zeng et al., 2018).

## 7 Future directions: multi-omics approaches to optimize TCM interventions through gut-host interaction

As scientific understanding deepens, integrating TCM with multi-omics technologies opens new frontiers in enhancing therapeutic precision. TCM has shown remarkable potential in modulating gut microbiota and influencing host metabolism, especially in treating metabolic and neurological disorders. However, the interactions between gut microbiota, host metabolism, and TCM interventions are complex and dynamic. Traditional research approaches struggle to capture these intricate mechanisms, making multi-omics technologies indispensable for precise and individualized interventions. To bridge this gap, recent studies have started applying multi-omics technologies, including metabolomics and metagenomics, to better understand the therapeutic mechanisms of TCM in diseases like PSD (Feng et al., 2022; Wang et al., 2022; Meng et al., 2025).

**FIGURE 4:**
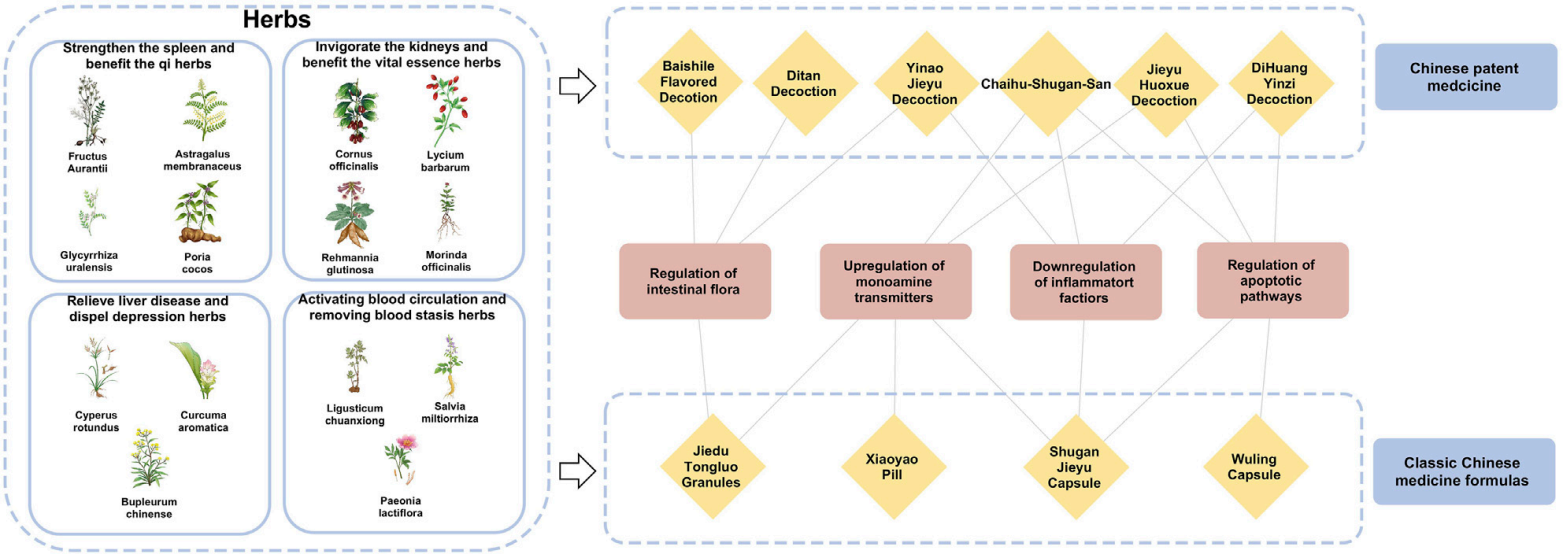
Herbal interventions for enhancing recovery in PSD.

Omics platforms such as metabolomics, metagenomics, proteomics, and single-cell omics offer new dimensions for understanding how active compounds in TCM reshape the gut microbiota and modulate biochemical pathways at various levels. These multi-layered insights allow researchers to unravel the intricate relationship between the gut and brain, identify key biomarkers, and optimize treatment strategies in diseases such as PSD (Figure 4). TCM’s active components—such as polysaccharides, flavonoids, and alkaloids—function by enhancing microbial diversity, supporting beneficial bacteria, and suppressing pathogens (Xia et al., 2022; Wang et al., 2024). Astragalus polysaccharides promote the growth of Lactobacillus and Bifidobacterium, while alkaloids in *Coptis chinensis* Franch. (Ranunculaceae; Huang Lian) inhibit pathogenic bacteria, thereby maintaining gut homeostasis (Bot et al., 2020; Du et al., 2021; Amin et al., 2023). These effects not only amplify the therapeutic impact of TCM but also encourage the production of key metabolites. Baicalin, for example, is transformed into baicalein by gut bacteria, enhancing anti-inflammatory and neuroprotective effects (Du et al., 2021). Similarly, ginsenosides from ginseng are metabolized into rare bioactive forms that improve glucose metabolism and reduce inflammation (Bot et al., 2020).

In patients with PSD, disturbances in the gut microbiota and alterations in metabolic pathways can exacerbate depressive symptoms. One significant mechanism is the shift in tryptophan metabolism towards the kynurenine pathway, which reduces serotonin levels, potentially intensifying mood disorders. Research has demonstrated that *S. miltiorrhiza* Bunge (Lamiaceae; Dan Shen) effectively restores metabolic balance by enhancing butyrate production, which offers both anti-inflammatory and neuroprotective effects, thereby alleviating symptoms of PSD (Badini et al., 2022). Further, the integration of metabolomics and metagenomics has deepened our understanding of how gut microbiota affects neurotransmitter production, energy metabolism, and immune function. This multi-omics approach provides a framework for personalized therapeutic strategies by adapting TCM interventions to individual microbial and metabolic profiles. Specifically, formulations containing *S. miltiorrhiza* Bunge (Lamiaceae; Dan Shen) and Astragalus membranaceus have been proven to regulate neurotransmitter balance, thus improving gut-brain communication.

Metabolomics offers insights into how TCM compounds modulate metabolic pathways associated with neurotransmitter synthesis, energy metabolism, and inflammation. For instance, Astragalus polysaccharides promote the growth of Lactobacillus and Bifidobacterium, while alkaloids in *C. chinensis* Franch. (Ranunculaceae; Huang Lian) inhibit pathogenic bacteria, thereby maintaining gut homeostasis (Bot et al., 2020; Du et al., 2021; Amin et al., 2023). In PSD, disruptions in gut microbiota and altered metabolic pathways exacerbate depressive symptoms. A key mechanism involves a shift in tryptophan metabolism toward the kynurenine pathway, reducing serotonin levels and intensifying mood disorders. Metagenomics deciphers the structural and functional composition of gut microbiota in response to TCM interventions. Research has shown that ginsenosides from *Panax ginseng* C.A.Mey. (Araliaceae; Ren Shen) are transformed by gut microbiota into rare bioactive metabolites, which enhance glucose metabolism and suppress neuroinflammation (Bot et al., 2020).Transcriptomics and proteomics allow for the exploration of gene expression changes and protein-level modifications triggered by TCM therapies. Baicalin, a flavonoid from *S. baicalensis* Georgi (Lamiaceae; Huang Qin), is metabolized by gut bacteria into baicalein, which enhances anti-inflammatory pathways and promotes neuronal survival (Du et al., 2021). These effects not only amplify the therapeutic impact of TCM but also encourage the production of key metabolites. Proteomic studies have also identified that Danshenextracts regulate neurotransmitter-related proteins, particularly those involved in serotonin and dopamine signaling, which are disrupted in PSD. Single-cell omics provides unprecedented resolution in identifying cellular heterogeneity within the gut-brain axis, revealing how specific immune cells, neurons, and glial cells respond to TCM-derived compounds. By mapping cellular interactions at the single-cell level, researchers can decipher the precise molecular targets of TCM therapies, refining treatment strategies for PSD and other neurological disorders. (Badini et al., 2022).

Multi-omics techniques elucidate the complex interactions between the gut and brain, laying a foundation for precision medicine in PSD. These methods facilitate personalized treatments by integrating TCM with contemporary diagnostic tools, enhancing TCM’s capacity to regulate inflammation, neurotransmitter functions, and metabolic processes, thus supporting early diagnosis and tailored treatments for PSD patients.

## 8 Conclusion and perspectives

PSD affects over one-third of stroke survivors, driven by complex factors like genetic predisposition, neurotransmitter imbalances, neuroinflammation, and gut-brain axis disruptions. While conventional treatments are effective for some, side effects and drug resistance highlight the need for alternative approaches. TCM offers a holistic strategy, targeting neurotransmitter regulation, neuroprotection, neuroinflammation, and gut microbiota modulation.

However, its clinical application faces challenges, including lack of standardized dosing, quality control variability, potential herb-drug interactions, and limited large-scale randomized controlled trials (RCTs). Additionally, integrating TCM with multi-omics technologies remains complex, requiring standardized methodologies to bridge traditional knowledge with modern precision medicine.

Future research should focus on standardized clinical trials and molecular mechanisms, including neurotrophic factors and microbial interactions. By addressing these challenges and leveraging multi-omics technologies, TCM can complement conventional therapies, optimizing recovery and improving quality of life for stroke survivors.
